# Cancer of Unknown Primary Site: Real Entity or Misdiagnosed Disease?

**DOI:** 10.7150/jca.42880

**Published:** 2020-04-06

**Authors:** Alaa T. Alshareeda, Batla S. Al-Sowayan, Reem R. Alkharji, Sahar M. Aldosari, Abdullah M. Al subayyil, Ayidah Alghuwainem

**Affiliations:** 1 Stem Cells and Regenerative Medicine Unit, Cell Therapy & Cancer Research Department, King Abdullah International Medical Research Center, King Abdulaziz Medical City, Ministry of National Guard Health Affairs, Riyadh 11426, Saudi Arabia; 2 Research Department, Health Sciences Research Centre, Princess Nourah bint Abdulrahman University, Riyadh, Saudi Arabia.; 3 Cytogenetic and Molecular Genetics, Prince Sultan Military Medical City, Riyadh, Saudi Arabia.

**Keywords:** Cancer of unknown primary, Immunohistochemistry, Genetic abnormality, Cancer Stem Cells, and angiogenesis

## Abstract

Metastasis is a late event in the progression of any tumour. However, invasive cancers are occasionally detected in the form of metastatic lesions without a clearly detectable primary tumour. Cancer of unknown primary site (CUP) is defined as a confirmed metastatic tumour, with unknown primary tumour site, despite the standardized diagnostic approach that includes clinical history, routine laboratory tests, and complete physical examination. Due to the lack of basic research on its primary causes, CUP is appropriately termed an 'orphan' cancer. Nevertheless, CUP accounts for 2-5% of diagnosed malignancies. To date, it is unclear whether CUP is an entity with primary dormancy as its hallmark or an entity with genetic abnormalities that cause it to manifest as a primary metastatic disease. In this review, we discuss different aspects of CUP, including its current diagnostic methods, angiogenesis effectors, relationship with cancer stem cells and current treatments.

## Introduction

Cancer of unknown primary site (CUP) is defined as metastatic cancer, where the primary tumour is undetectable with the standardized diagnostic approach that includes clinical history, physical examination and routine laboratory tests 1, 2.

Despite significant advances in cancer diagnostic tools, the American Cancer Society estimated that around 31,480 patients would be diagnosed with CUP, which represent about 2-5% of diagnosed cancer patients 3. Worldwide, CUP remains the 6^th^ to the 8^th^ most common cancer, and the 3^rd^ to the 4^th^ most common cause of cancer-related deaths 4, 5. The clinical presentation of CUP is heterogeneous; 42 % of patients seek medical attention due to enlargement of a superficial lymph node. However, 33% of patients have symptoms related to metastatic lesions in the liver, 26% in lung, 22% in bones, 9 - 11% in the mesothelial lining or metastases may be detected by chance during clinical radiology for other diseases 6.

The debate about the clinical implications could affect the treatment choices. This raises the importance of having a biological molecule to identify the tumour. There are two hypotheses to explain the biology of CUP; one suggests that a given tumour can develop without a premalignant lesion or primary tumour; that CUP has a clear genetic and epigenetic identity and raise the need of identifying the molecular signature in the level of chromosomes 7. The other hypothesis postulates that CUP is an artificial classification of malignant metastatic tumour, as the metastasis develops early on in the disease process, as the primary tumour and its metastases progress in parallel. Gene profiling assays could classify that type. However, it could generate confusion between CUP and metastases of known origin 8.

Epidemiological studies revealed that the median age of CUP diagnosis is ~60 years with no significant difference in incidents between the sexes 9. Only 15-20% of patients diagnosed with CUP exhibits favourable features, whereas the remaining patient population display an aggressive form of the disease with an unpredictable pattern of metastatic spread and resistant to standard chemotherapy, leading to reduced survival rate (median of 5-11 months) 10.

This review aims to highlight different aspects around CUP such as diagnostic methods, angiogenesis effectors, association with cancer stem cells and current treatments to draw a map for researcher and clinician to support research in this area of interest and improve patient outcome.

## Pathophysiology and metastasis of CUP

The guidelines issued by the United Kingdom National Institute of Clinical Excellence (NICE) for the management of CUP provided for the classification of definitions replicating the different phases of the investigation (Table 1).

The pathophysiological basis for CUP is still ambiguous; the origin of the primary tumour cannot be identified, even in an autopsy setting. Different hypotheses have been suggested to clarify the existence of this clinical entity 11, 12. One such hypothesis considers the CUP to be an early metastatic presentation of primaries with a dominant metastatic phenotype. In such instances, metastatic tumours may develop before the primary tumour became large enough to be detected on imaging studies. In a few cases, the primary tumour appears during or after treatment; these cases are referred to as "latent primaries".

Another relevant dispute is whether the prognosis of CUP patients is linked to the prognosis of the primary tumour or a genetic profile typical to CUP. Table 2 shows the main factors associate with a poor prognosis of CUP. The metastasis-prone behaviour of CUP may be related to a functional deficiency of certain metastasis-suppressor or tumour-suppressor genes. Identifying them will help to dispel the belief that dysregulation of one or more genes and their encoded proteins pushes systemic dissemination and primary regression. Klein et al. 13 have suggested that neoplasm might develop from stem cells, without triggering a premalignant lesion or a primary tumour. These basic hypotheses have not been confirmed because studies conducted to date have yielded neither consistent nor specific gene/protein abnormalities 'pivotal' to the development and survival of CUP 14. Table 3 summarizes some suggested reasons for the difficulty in determining the primary site of the tumour.

CUP is considered to be an aggressive metastatic disease, but it is not known whether the prognosis is different from the metastatic cancers of known primary site (Table 4). The unpredictable metastatic pattern in diagnosis refers to variations in the incidence of metastatic sites between known and unknown primary cancers, i.e. pancreatic cancer presenting as CUP has a 4-fold higher incidence to affect bones and a 30% incidence of lung metastases compared to the known natural history of known primary pancreatic cancer 1. The aggressive behaviour of CUP may be due to initial immunosuppression, which may lead to mutation accumulation. The unchecked spread of tumour occurs upon escape from the suppressed state 15. Although the primary tumour in CUP is thought to be dormant, CUP patients have early distant metastases 16. The metastatic tendency may explain poor prognosis, and metastasis is considered to be the cause of death in most patients with primary cancers 17. Metastasis involves several genes, and it has been shown that some essential metastatic genes are overexpressed in CUP: vessel endothelial growth factor 18, and matrix metalloproteinases, proteolytic enzymes that mediate local invasion and metastasis 19.

The pattern of CUP spread at diagnosis may provide indications as to whether the primary site is above or below the diaphragm. Metastases of the liver are more frequent from primary disease under the diaphragm. The pattern of carcinoma metastases presented as CUP may be considerably different from that predicted from the usual presentation. For example, bone metastases are approximately three times more frequent in pancreatic cancer that is present as CUP. In contrast, osseous lung cancer metastases are about ten times less common when revealing as a CUP compared to the usual presentation 20.

## Diagnosis of cancer of unknown primary site

**Figure 1:**
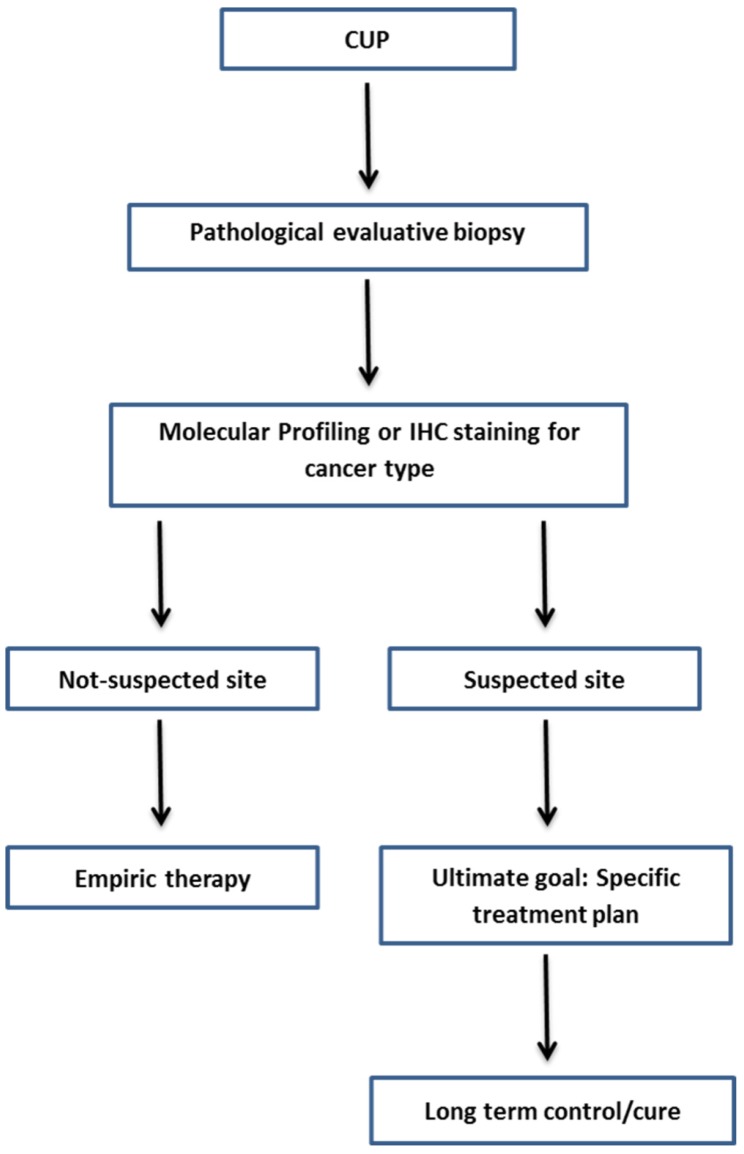
Framework diagnosis of CUP.

A CUP diagnosis is often reached when the patient has a histologically confirmed metastatic cancer with unidentifiable primary tumor site, despite the standard diagnostic approach (Table 5) 4. It is believed that identifying the site of primary tumour will improve the customization of therapy, results in improvement of the patient's survival rate. Along with the complete physical examination and medical history, laboratory and radiological examinations, traditional Immunohistochemistry (IHC) methods and molecular-based assays are applied to identify the tissue of origin for CUP. Figure 1 shows the current framework of the CUP.

### Immunohistochemistry

IHC and biomarkers study is a useful method to evaluate the primary tumor histology in patients with CUP. The investigation of CUP's tissue by IHC comes in a three-step; first looking for the wide type of cancer such as carcinoma, sarcoma, melanoma and/or lymphoma. Then identify the subtypes such as adenocarcinoma, germ-cell tumor, hepatocellular, renal, thyroid, neuroendocrine, and/or squamous carcinoma. Lastly, give information about the primary site of cancer as an example: prostate, lung, breast, colon, pancreas or biliary, or ovarian cancer 21.

Initially, IHC and light microscopy is employed to classify CUP into one of five subtypes. These subtypes are; well- or moderately differentiated adenocarcinoma (60%), poorly differentiated adenocarcinoma or undifferentiated carcinoma (29%), squamous-cell carcinoma (5%), poorly differentiated neoplasms (5%), and neuroendocrine tumors (1%) 22. Then, IHC is further employed in order to identify the primary site. The most commonly added proteins to the IHC CUP staining panel are the keratin family members; CK7 and CK20, with the CK7^+^/CK20^-^ being the most common in CUP (CK7^+^/CK20^-^ Breast, Ovarian, Pulmonary, Endometrial, Thyroid; CK7^+^/CK20^+^ Upper gastrointestinal, Pancreatic, Urothelial; CK7^-^/CK20^+^ Colorectal, Merkel cell; CK7^-^ /CK20^-^ Prostatic, Hepatocellular, Renal cell, and Adrenal cortical) 23. Other cytoplasmic markers that are commonly added to the IHC staining panels also include; SCGB2A2 and GCDFP-15; breast origin, TTF-1; lung origin, HepPar-1; liver origin, RCC and PSA; renal and prostate origin respectively 24. It is estimated that using the IHC staining panels is enough to identify the primary site in about a third of diagnosed CUP cases 25. The major limitation of IHC staining is that IHC is not useful in the case of poorly differentiated cancers in addition to the inability to get an adequate biopsy sample and the variable interpretation by different pathologists. Nevertheless, it has been shown that IHC approach is the most effective in detecting the primary site in some cases, including non-small cell lung, colorectal, breast, and ovary or kidney cancers. Whereas other cancer types, such as the pancreas, gastric, biliary and urothelial cancers, are less suited for IHC stating and requires other means of detection 26.

### Molecular-based assays

Gene expression profiles (GEP) have demonstrated a higher accuracy (91%) than IHC (71%) for poorly differentiated and undifferentiated carcinomas 27. Currently, there are many commercially available GEP-based assays for CUP, such as Veridex, Agendia, bioTheranostics Inc., [real-time polymerase chain reaction (RT-PCR) for mRNA], Rosetta Genomics Laboratories, Philadelphia, PA and Prometheus Laboratories, San Diego, CA (RT-PCR for microRNA) and Pathworks Inc., Redwood City, CA, USA (microarray for mRNA; Table 6). The claimed accuracy of these tests to predict the primary site of CUP compared with IHC and/or autopsy ranges from 78-88.5%. It should be noted that some of these assays display reduced accuracy for poorly differentiated tumours or specific tumour types such as lung and pancreatic cancers. This is challenging because these two sites are the most prevalent primary cancer sites in the diagnosed CUP cases 28. Differences in the biological functions of each tissue may explain the variety of GEP evident in CUP. During carcinogenesis, the conservation of a tissue-specific GEP may assist in the characterisation of CUP and its primary site. However, the 1550-gene microarray-based Pathwork Tissue of Origin Test (Pathwork Diagnostics Inc., Redwood City, CA, USA) is the only test approved by the United States Food and Drug Administration 29, 30.

Biologically, CUP is classified using tissue microarrays: the expressions of multiple genes in samples of known primary tumours are compared with the GEP of patients with CUP. Many studies have also used (RT-PCR) to classify CUP. Using RT-PCR, the expression of 10 signature genes in 120 patients with CUP was examined and revealed a putative tissue of origin in 61% of the patients 29, 31. Another study that used a similar method to investigate 92 genes showed an overall accuracy of 82% amongst 39 types of cancer 32. Besides, RT-PCR was used to evaluate 20 patients with CUP, the diagnoses of 15 patients was proven to be corrected when the primary tumours were identified during autopsy 30.

It has been previously shown that the mutations of somatic point identified in a tumor can be utilized to identify its site of inception with restricted precision. Marquard *et al.* have 33 hypothesized that higher exactness could be accomplished by classification algorithm in light of the accompanying capabilities: 1) the quantity of non-synonymous point mutations in a set of 232 specific cancer-associated genes, 2) frequencies of the 96 classes of single-nucleotide substitution dictated by the flanking bases, and 3) copy number profiles, if accessible. They have created and analyzed the execution of characterization calculations using different composes and measures of data. Recognizable proof of primary site from point mutation as well as copy number information might be sufficiently precise to help clinical analysis of CUP. In particular, they have estimated that copy number profiles would add to the classifier execution 33. In any case, although tumor copy number profiles can be obtained from entire genome or entire exome sequence data 34, the quality and unwavering quality relies upon sufficient sequencing profundity, which is not accessible for all sequenced samples. Subsequently, they have assessed classifiers based on somatic point mutations types, including; single nucleotide substitutions, short inclusions and deletions. Alternatively, they have assessed classifiers based on point changes or addition in SCNAs, independently.

### Molecular biology of cancer of unknown primary site

A significant effort is being made to detangle the CUP's molecular characteristics. Understanding the genetic mechanism behind the disease will enable clinicians to better customize a treatment for this disease. Different cancers present different GEP based on the normal GEP of the tissues of the primary site. However, several studies have identified several molecular features that are shared by all CUP cases. Pentheroudakis et al. 35 investigated biopsies collected from women with CUP involving either the axillary nodes or peritoneum. These biopsies were compared to reference samples from women with breast or ovarian cancers, respectively 35. No differences in GEP were identified using the 64-microRNA assay.

### Genetic abnormality

Aneuploidy is detected in 70-90% of solid tumours and likely reflects aberrant chromosomal replication during cell division. Aneuploidy is seen in 70 percent of patients with abnormalities in the short arm of chromosome 1(1p), and chromosome 12 have been identified. The existence of isochromosome12, i(12p) or deletion in 12p describes the germ cell origin tumour and represents patients with midline nodal CUP metastases 36. In 1991, Motzer *et al.* 37 reported the presence of an extra copy of the short arm of Chromosome 12, i(12) in 12 of 40 patients with CUP. The presence of this marker predicted for a response to cisplatin-based chemotherapy 38. Furthermore, Pantou *et al.* 39 analysed 20 CUP cases and showed cytogenetic patterns, with abnormal karyotypes. Multiple chromosomal rearrangements were detected (15 changes) involving mostly chromosomes 1, 3, 6,7,11, while others were characteristic of different histological subtypes (4q31, 6q15, 10q25 and 13q22; more frequently encountered in adenocarcinomas). Additionally, complex karyotyping is a prognostic for worse survival compared to patients with up to five alterations.

### Oncogenes and proteins expressed by cancer of unknown primary site

Oncogenes are genes that, when dysregulated or activated have the potential to develop cancer. The molecular basis of chromosome instability in sporadic cancers remains poorly understood, but the collapse of DNA replication forks caused by oncogenes, leading to DNA double-strand break and genomic instability, is considered an appealing model 40. The transformation and survival of malignant cells involve many cellular processes, including proliferation, migration, inhibition of apoptosis and promotion of neoangiogenesis, all of which are activated by the encoded proteins 14.

The roles of Epidermal Growth Factor Receptor family (EGFR) have been extensively studied in CUP 41-43. Table 7 shows the available data for the alterations of tyrosine kinase with different percentages in different CUP studies. These inconsistent results demonstrate the heterogeneity of the CUP. PCR failed to identify mutations in Exons 18, 19 or 21 of EGFR in any of the 50 tumours 43. Ross *et al.* (2015) performed comprehensive genomic profiling for 200 patients with CUP and showed that mutations of *human epidermal growth factor receptor 2* (*HER-2*) were more frequent in adenocarcinomas of unknown primary site (13 patients [10%]) than in non-adenocarcinomas of unknown primary site (3 patients [4%])^44^. Besides, alterations in *EGFR* (10 patients [8%]) were more common in adenocarcinomas of unknown primary site than in non-adenocarcinomas of unknown primary site. The lack of markers of active EGFR signalling in CUP could indicate a lack of value for EGFR modulation 43.

Preclinical and clinical studies have proved that constitutive activation of *C-KIT* is an early, essential and sufficient oncogenic stimulus for malignant transformation and gastrointestinal stromal tumours remain dependent on it for continuing growth 45, 46. In addition, *platelet-derived growth factor (PDGF)* expression has been linked to the development of many cancers, such as gliomas and gastrointestinal stromal tumours 47, 48. Dova *et al.* (2008) studied C-KIT and the PDGF receptor (PDGFR) in a moderately-sized cohort of patients with CUP (*n* = 50) and found that overexpression of these two proteins is rare and has no gross prognostic significance for survival and no association with the presence of activating mutations 49.

### Tumour suppressor genes/proteins involved in cancer of unknown primary site

Tumour suppressor genes, or anti-oncogenes, are genes that encode proteins that suppress malignant transformation, survival and metastatic dissemination, protecting cells from becoming cancerous by preserving the integrity of cellular DNA and regulating vital cell-cycle processes in combination with the 'genome guardian', *P53*. Many tumour suppressor genes have been investigated, but only *P53* and retinoblastoma (*pRb*) are widely understood. Table 8 shows the tumour suppressor genes or proteins in CUP. Briasoulis *et al.* 50 investigated P53 expression in 47 patients with CUP using IHC. 70% of the patients showed positive staining for P53, whereas 53% of them exhibited a high immunoreactivity index for P53. Bcl-2 is known to function downstream of P53 51. Briasoulis *et al*. reported 65% and 40% of Bcl-2 expression and overexpression, respectively, in CUP patients. Only 20% of the patients had both Bcl-2 and P53 overexpression. Notably, detection of either protein was not associated with any of the major clinicopathological parameters.

In 15 patients with CUP and eight-cell lines established from CUP, Bar-Eli *et al*. 52 studied the frequency of *P53* Exon 5-9 mutations by direct DNA sequencing of PCR products. Only 26% of tumours showed mutated *P53,* suggesting a relatively low frequency of *P53* mutations in CUP. In contrast*,* Ross *et al.* 44 showed that the most frequent genomic alteration was in *P53* (55%) followed by Breast Cancer susceptibility gene* (BRCA2)* (6%), after comprehensive genomic profiling of 200 patients with CUP. Additionally, Gatalica *et al*. 53 investigated 1806 patients with CUP and showed that *TP53* was the most commonly mutated gene. However, this discovery did not help to identify the site of origin 4.

The Phosphatidylinositol 3-kinase- Protein kinase B/ Phosphatase and tensin homolog (PI3K-Akt/PTEN) pathway is involved in the initiation, progression, cell growth, proliferation, metabolism and survival in cancer 54. The frequency of inactivation of *PTEN* in somatic cancer is high, and it is ranked the second most mutated tumour suppressor gene after *P53* 55. To date, efforts to develop targeted therapies have been unsuccessful for reasons including extensive internal intra-pathway or external inter-pathway negative feedback loops or networking between pathway suppressors. The tumour suppressor *PTEN* is considered the main brake for this pathway and has attracted extensive interest as a target for inactivation in somatic cancers 56. Golfinopoulos *et al*. 57 studied the roles of the phosphorylated active forms of Akt and PTEN in 100 patients with CUP using IHC. PTEN and phosphor- AKT were overexpressed in 60% and 85% of these patients, respectively. Comprehensive genomic profiling performed by Ross et al. 44 identified genomic alterations in *PTEN* in 200 (7%) of patients with CUP.

The *BRCA1* and *BRCA2* tumour suppressor genes play an important role in DNA damage repair to prevent the development of tumours. Mutations in these genes confer a high risk of breast and ovarian cancers 58. *BRCA1* mutation carriers are at high risk of CUP (relative risk [RR] = 3.45, 95% confidence interval [CI] = 2.35-5.07, *P* < 0.001) 59. Mersch *et al*. 60 completed an institutional review board-approved study and identified deleterious mutations in *BRCA1* and *BRCA2* in four patients with CUP.

Metastasis is a complex process that involves both metastasis-promoting and metastasis-suppressing genes 61, 62. *Kisspeptin1 (Kiss-1)* has been identified as a human metastasis-suppressing gene with the ability to suppress the metastasis of certain cancers, such as melanoma and breast cancer 63. Dova *et al*. 64 showed that only one patient harboured a mutation in *Kiss-1* out of fifty patients with CUP using PCR- single-strand conformation polymorphism (PCR-SSCP) and direct sequencing.

The different specificities of antibodies may explain the discordance between IHC data and data obtained from the mutational analysis of genes for wild-type and mutated genes. For example, the varying impact of *P53*-regulating factors such as murine double minute-2, p14 alternate reading frame and p21 due to presence of mutations outside Exons 5-9 of *p53* 65, 66.

## Angiogenesis of cancer of unknown primary site

Angiogenesis is the process of new blood vessel formation, and tumours rely on it for growth beyond a size of 3-4 mm, survival and invasion 67. Vascular endothelial growth factor (VEGF) is considered the key molecule that facilitates the proliferation of endothelial cells 68. The lack of angiogenesis in primary tumours, inducing dormancy, but the presence of angiogenesis at metastatic sites may represent a model to explain the biology of CUP. However, studies have indicated that VEGF expression is not associated with prognosis, except for a positive association between VEGF and the density of micro-vessels (indicated by markers such as Cluster of differentiation; CD34) 69, 70. Van de Wouw *et al*. 69 supported this using IHC by showing no prognostic effect of CD34 and VEGF on the outcomes of patients with CUP, although VEGF was overexpressed in 26%.

Similarly, Hillen *et al*. 71 showed no differences in the density of micro-vessels in 39 liver metastases from patients with CUP and 30 liver metastases from colon and breast cancer: both groups exhibited high angiogenic activity. Another study by Agarwal *et al.* 70 reported low expression of VEGF protein in patients with CUP, where 50 patients with squamous carcinomas metastatic to the cervical lymph nodes were compared with 52 patients with metastases from a known primary. Also, they proposed a pattern of metastatic spread for squamous CUP metastasizing to the cervical lymph nodes independent of angiogenesis 70. Karavasilis *et al*. 72 reported that patients with unfavourable CUP showed higher angiogenetic activity than those with favourable CUP (70 vs 46 microvessels/mm^2^).

## Cancer stem cells and cancer of unknown primary site

The hypothesis of cancer stem cell (CSC) suggests that these cells are accountable for maintaining tumour heterogeneity, operating tumour growth and resistance to therapy. There is a challenge in discrepancies between CSCs and normal stem cells because of the limitation in purification techniques. The biomarkers remain the same in most cases of characterization, and the key of differentiation is the function 73.

Sell and Pierce (1994) proposed that a cancer cell arises as a result of stem cell mutation rather than somatic cell differentiation 74. In their proposal, they claim that neoplasia occurs in stem cells, whereas hyperplasia occurs in somatic cells. Most solid tumours are of unknown cellular origin, and the variety of these tumours is believed to reflect different cells of origin.

The stem cells' long lifespan and ability to self-renew support the idea of malignancy arising from these cells. Some studies have proposed that the biological events that occur during metastasis resemble the stem cell-based processes that occur during embryonic development. Cells undergo many phases during development, including division, migration and specialization. Stem cells (such as embryonic and mesenchymal stem cells) can travel long distances, invade and engraft into the targeted tissue, then differentiate into tissue-specific cell type 75. This ability of stem cells to migrate is suppressed following embryonic development, but this ability most likely return in pathological conditions. Therefore, the ability of stem cells to migrate from their original site into new tissues may contribute to the phenomena of CUP. The migration of cancer/affected stem cells from their tissue of origin into host tissue may lead to the formation of cancers at a new site earlier than, or without the development of, a tumour in the original tissue. Besides, it is essential to understand that cancer development in any tissue is not a prerequisite for stem-cell to migrate from that tissue. This theory may explain why the primary tumour site is not detected in some CUP diagnosed patients, even with a post-mortem examination. Invasion of the surrounding tissue is considered one of the initial steps in tumour cell migration. This invasion is achieved by the secretion of proteolytic enzymes, such as matrix metalloproteinases (MMPs; MMP2 and MMP9). Karavasilis *et al.* (2005) investigated the expression of these two enzymes in 75 CUP patients and found that they were overexpressed in 49% and 36% of CUP cells, respectively, but not in the stroma 76. Furthermore, Kamposioras *et al.* (2013) showed the absence of stem-cell markers CD133 and octamer-binding transcription factor-4 (OCT4) in 100 CUP tumour samples by using IHC. Conversely, CUP cells circulating in peripheral blood showed positive expression of aldehyde dehydrogenase-1 as visualised by immunofluorescence (*n* = 7/14) 77. Thus, stem-cell phenotype acquisition by CUP may be an event that is infrequent, transient or dynamic.

Tyrosine-protein kinase or hepatocyte growth factor receptor (Known as* HGFR or MET)*, a proto-oncogene expressed in both stem and cancer cells, is a crucial regulator of invasive growth 78. Stella *et al.* 79 found an extremely high incidence of *MET* somatic mutations located in nucleotides clustered, in either the kinase or extracellular semaphorin domains, in 50 patients with CUP. The mutated receptors remained functional and sustained the transformed phenotype, implying that *MET* activating mutations are genetic biomarkers related to CUP. Consequently, the mutation of *MET* may indicate the grade of differentiation and/or original organ. Accordingly, favoured expression of *MET* in cancer stem cells has been proposed 78**.**

## Treatment of cancer of unknown primary site

Over the last 20 years, the treatment of patients with CUP has progressed slowly. Rather than continuing to test existing chemotherapeutic regimens, most clinical trials involving patients with CUP have focused on the development of improved diagnostics to facilitate accurate prediction of the primary site. Table 9 summarises the therapeutic options according to the European Society of Medical Oncology guidelines 80. Almost 20% of patients with CUP express clinical and/or pathologic characteristics that classify them into one of several known 'treatable subsets”. Efficient chemotherapeutic agents were non-specific, and development of a 'broad-spectrum' combination with good activity against highly sensitive tumour types was desirable 26. The treatment of unfavourable subsets of patients with CUP (80%) mostly comprised empirical chemotherapy with platinum or taxane combinations 81. Unfortunately, the response to treatment is low (around 20%). A comparative review of survival and chemotherapy regimens for CUP by Golfinopoulos *et al.* concluded that no type of chemotherapy had demonstrated any survival benefit in these patients 82.

The incorporation of site-specific treatment achieved using remodelled diagnostic methods may allow the evaluation of particular molecular abnormalities depending on the prediction of the primary site. For example, more focused investigations including tests for activating mutations of *EGFR* and re-arrangements of *Anaplastic lymphoma kinase* (*ALK*) and *proto-oncogene receptor tyrosine kinase ROS1* are evident in patients with CUP in the form of non-small cell lung cancer. Penley *et al.* 83 revealed the efficacy of crizotinib (an ALK inhibitor) in a small group of patients with non-small cell lung cancer with positive ALK re-arrangements.

Whatever the prognostic significance of angiogenesis in CUP, the first tests of treatment using anti-angiogenic agents yielded promising results. The combination of bevacizumab and erlotinib has been investigated in first-line (in combination with chemotherapy) and second-line Phase-II studies 84, 85. In the first-line therapy, the disease was controlled in 82% of patients, with progression-free and overall survival rates of 8 and 126 months, respectively 84. In the second-line treatment, two targeted agents were combined (excluding chemotherapy), achieved an increase in the rate of clinical benefit of 71% and a median overall survival of 74 months. These two agents were well tolerated, although it needs further confirmation in Phase-III trials before drawing a solid conclusion. Although these characteristics are not that different from those seen in advanced CUP, they deliver a sound basis for the use of anti-angiogenic therapy combined with anti-proliferative therapy in clinical settings.

Site-specific treatment plans in patients with CUP patients based on predictions by GEP and/or IHC provide an improvement in the overall outcomes of patients; patients expected to show treatment-sensitive tumour types experience with the most significant benefit. In case IHC staining fails to predict a single primary site, a GEP assay should be included in the diagnostic plan for the evaluation of patients with CUP. Finally, site-specific treatment, based on the diagnosis of the primary tissue, must replace empirical chemotherapy in patients with CUP 26.

## Discussion

Although there are biomarkers for different types of cancers, there are no specific and predictive biomarkers for the metastatic phenotypic spectrum 86. A logical way to address this is to understand the complex biological steps that occur during metastasis. Regarding the oncogenic molecular assets of CUP, numerous studies have assessed the expression and mutation status of both oncogenes and tumour-suppressor genes. Surprisingly, lesions of the leading players recognized to drive most human cancers cannot be identified in CUP. In theory, suppress apoptosis and increased cell survival triggered by aberrant EGFR signalling would allow CUP cells to escape cell death, accumulate genetic damage and develop an early metastasis phenotype.

Most of the genetic aberrations present in CUP are due to the 1p chromosomal deletion 87. One hypothesis is that a tumour-suppressor gene for metastasis is located on this chromosome (1p); therefore, its alteration leads to high propensity for metastasis, although it is subject for further investigation 88. Further validation of these biomarkers in large prospective trials is necessary to plan rational trials for the optimization of CUP treatment.

Previously, GEP analyses facilitated the biological assignation of some patients with CUP to different primary sites, but biological variations between CUP and typical metastatic solid cancers could not be detected. Investigators interested in CUP must improve their research by including patients with CUP with poor prognosis in epigenetic, proteomic and microRNA studies to further explore the complexity of CUP. Epigenetics must also be examined similarly to the recent study by Moran *et al.* 89, who created a tissue of origin molecular profiling assay based on the methylation status of DNA. Furthermore, a study identified primary sites in 87% of the patients tested, compared to those of the Pathwork Diagnostics 90 and BioTheranostics Cancer Type ID 91 assays. Using such approaches may solve practical issues such as tissue availability and cost compared with IHC or RNA-based assays. Ideally, patient autopsies must be considered during the validation of tumour of origin. However, given legal and ethical issues, these studies are very challenging in current clinical practice.

Different examinations have tended to the critical issue of deciding the primary site of tumours by molecular profiling. However, most past reports have utilized gene expression profiles from microarrays 92 or quantitative PCR 91, or in a couple of cases microRNA expression profiling 93, 94. It is a perceived issue that gene expression-based classifiers do not perform well on a poorly differentiated tumour sample, apparently because of the changes in gene expression drive differentiation. Noteworthy wellsprings of circulating microRNAs are blood cells, and the levels of numerous detailed tumour circling microRNA biomarkers relate to blood cell count 95, 96. Likewise, genomic profiling gives a stronger and malignancy particular estimation, which primarily depends on DNA as opposed to RNA for tumour characterization.

It is accepted that metastatic lesions usually share their degree of differentiation with the primary tumour, so after the acquisition of a proliferative phenotype and colonisation of a distant organ, cancer cells may undergo a programmed mesenchymal-epithelial transition to re-adopt an earlier state of differentiation. In this cellular plasticity model, the concept of traveling cancer stem cells has been proposed to describe the different states and their interactions 97. Histological analysis of some patients with CUP reveals the presence of poorly differentiated or undifferentiated cells. It has been suggested that undifferentiated cells of these tumours represent CSC. The CSCs have been distinguished by their aggressive behaviour and play critical role in resistance to therapy. Therefore, anti-CSC treatment may work effectively in a large number of patients with CUP.

Because CUP has no existing standard therapy, it represents a unique opportunity to use comprehensive genomic profiling to lead targeted therapy as an initial treatment. Knowledge of the genomic alterations present in CUP is growing rapidly, and any mutation that is not clinically relevant today may be relevant in the future. Therefore, comprehensive genomic profiling will improve the outcomes of patients with CUP by facilitating the precise application of targeted therapies.

## Conclusion

**Figure 2:**
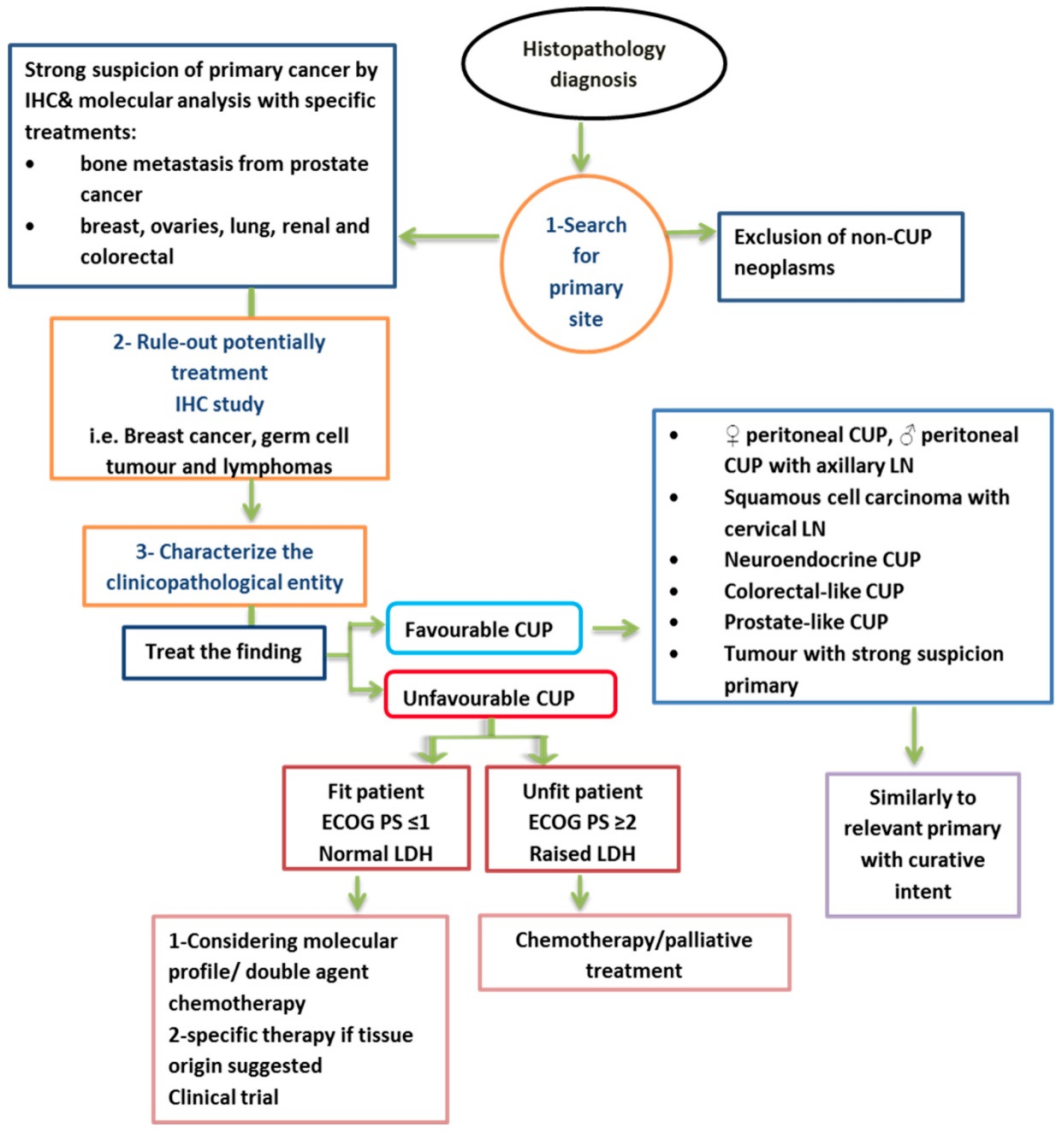
Summary of proposed management of CUP 1. LN= lymph node, ECOG= Eastern Cooperative Oncology Group

In summary, many clinical trials suggest that most human cancers arise due to a hit in normal stem cells. The migration of stem cells (deregulated premalignant or cancerous stem cells) from their original site to other locations may give rise to cancer in new locations before or without the development of a tumour in the original tissue. Site-specific therapy must be considered to increase treatment specificity for many types of advanced CUP. This will be achieved by improving diagnostic methods such as IHC staining and GEP, which leads to accurate prediction of the primary site in most patients 26. CUP with a unique molecular profile of primary cancer is most likely biologically different from primary tumours. Currently, no molecular profiling test can replace a clinically identified primary tumour. Figure 2 shows a summary of a proposed management of CUP.

## Figures and Tables

**Table 1:** classification of CUP by the National Institute of Clinical Excellence (NICE)

Term	Definition
Malignancy of undefined primary origin	Metastatic cancer without clear primary site using limited investigation, before performing comprehensive tests.
Provisional CUP	Metastatic epithelial or neuroendocrine cancer without any primary site of origin using selected initial cytological and histologic analysis, before specialist evaluation and probable post further specialized tests.
Confirmed CUP	Final histology showed metastatic epithelial or neuroendocrine cancer without any primary site of origin, even after preliminary tests, specialized assessment, and probably further specialized investigations.

**Table 2:** Main factors associated with the poor prognosis of CUP

Factor	Explanation
Gender	Male more than female
Type of metastasis	Multiple brain metastases
Organ involvement^*^	Pleural/lung, Liver, and adrenal involvement
Histological type	Adenocarcinoma

^*^Conversely, involvement of the lymph node and neuroendocrine histology is correlated with a better survival

**Table 3:** Suggested reasons for the difficulty in determining the primary site of the tumour.

Reason	Explanation
Primary tumour size	Very small; hard to detect
Tumour growth	Slow
Immune system	The immune system of the body killed the primary cancer.
Surgery; tumour removal	During surgery, primary cancer was removed for another condition by doctors without knowing that cancer had formed.

**Table 4:** Comparison between metastatic cancer and CUP

Factors	Metastatic cancer	CUP
**Source of the cancer**	Always defined	Mainly never determined
**Classification**	it's named after the part of the body where it started	Cancer of unknown primary or occult primary cancer.
**Primary organ**	Known	Unknown
**Staging system**	TNM system is the most widely used; describes the size of the primary tumour, nearby involved lymph node, and distant metastasis.	No staging system exists for CUP, the staging depends on the histology of the cancer 98.
**Stage**	Mainly stage IV	All CUPs are at least a stage II, and most of them are stage III or IV. Although the precise stage of the patient with CUP may not be known certain assumptions about the prognosis depending on which organs are impacted by cancer can still be made.
**Type**	Has the same type of cancer cells as the primary cancer	The origin unknown, mainly are epithelial cells; Adenocarcinoma.
**Common organ**	The lungs, liver, brain, and bones	Most of the times three or more organs are involved
**Prognosis**	Poor prognosis	Poor prognosis
**Treatment**	The best treatment for metastasis is the treatment of the primary cancer. Therapies may include chemotherapy or hormone therapy, immunotherapy, radiation therapy, surgery, or a combination of these.	Some treatments are standard (the currently used treatment), and some are being tested in clinical trials.
**Survival**	Better survival than CUP, except those with brain and respiratory metastases 15	Median overall survival of 3-9 months. The favourable prognostic group may have a median survival of nearly 36 months 15, 99.

**Table 5:** Required investigations for searching the primary site 4.

Clinicopathological data	Work-up for all patients	Work-up for selected patients
Histologically confirmed metastatic cancer	Full blood count	Mammography (for all women) and breast MRI
Detailed medical history	Biochemistry	Testicular ultrasonography
Complete physical (including pelvic and rectal) examination	Urinalysis and occult blood in stools	PET or CT scan and endoscopy
Histopathology review with specific IHC study	Chest radiography and CT scan of thorax, abdomen and pelvis	Concentrations of serum α-Fetoprotein, β human Chorionic Gonadotropin, Prostate-Specific Antigen (for all men), Cancer Antigen 125 and Carcinoma Antigen 15-3

MRI; Magnetic resonance imaging. PET; positron emission tomography. CT; computerized tomography. IHC; Immunohistochemistry.

**Table 6:** Commercially available molecular assays using gene-expression profiling for cancer of unknown primary site

Supplier	Test	Platform	Material	No. of genes profiled	No. of tumour classes	Reference
**Pathwork Diagnostics**	ResponseDX Tissue of Origin™ Test	RNA extraction/ microarray	Fresh	2000	10	100
**BioTheranostics**	CancerTYPE ID^®^	RT-PCR	FFPE	92	54	30
**Rosetta Genomics-Prometheus**	miRview_ mets (ProOnc Tumour SourceDxT)	RT-PCR for microRNA	FFPE	48	42	101

RT-PCR; Reverse transcription polymerase chain reaction. FFPR; Formalin-fixed, Paraffin-embedded.

**Table 7:** Alterations of tyrosine kinase in different CUP studies

Marker	c-Myc	Ras	HER-2	EGFR	C-KIT
**IHC finding**	Expression 96% Overexpression 23%	Expression 92% Overexpression 23%	Expression 65-68% Overexpression 4-27%	Expression 74-75% Overexpression 4-61%	Expression 12-81% Overexpression 4-13%
**Reference**	102	102	41, 42, 102	41-43	42, 103

**Table 8:** Tumour-suppressor genes and proteins in cancer of unknown primary site

*n*	Method	Findings	Reference
40	IHC	P53; expression 70%, overexpression 53%	50
200	Genomic profiling Illumina HiSeq2500 instrument	Genomic alteration of TP53; 55%, BRCA2; 6%, PTEN; 7%	44
23	PCR-SSCP	Exon 5-9 P53 gene mutations; 26%	52
100	IHC	Expression of PTEN; 60%, and Akt; 85%	57
8	Genomic testing	BRCA1 mutation carrier (RR^*^ = 3.45, 95% CI = 2.35-5.07, P < 0.001)	59
4	An institutional review board-approved study (genomic testing)	Mutation of BRCA1, 50% and BRCA2, 50%	60
50	PCR-SSCP	One case with Kiss-1 Exon 4a, 242C>G mutation (P81R)	64

^*^RR = relative risk, PCR-SSCP= PCR- single-strand conformation polymorphism, IHC=Immunohistochemistry. n=number of patients

**Table 9:** Therapeutic options for cancer of unknown primary site according to the European Society of Medical Oncology 80, 104.

Tumour type of CUP	Treatment plan
Poorly differentiated neuroendocrine carcinoma	Platinum + etoposide combination chemotherapy
Isolated axillary nodal metastases	Axillary nodal dissection, mastectomy or breast irradiation and adjuvant chemohormonotherapy
Adenocarcinoma with a colon profile	Chemotherapy regimens for colorectal cancer
Single metastatic deposit from an unknown primary	Resection and/or RT ± systemic therapy
Unfavourable subsets	Platinum-based empirical chemotherapy

RT; radio therapy.

## Abbreviations

CUP: Cancer of unknown primary site
NICE: National Institute of Clinical Excellence
IHC: Immunohistochemistry
Magnetic resonance imaging: MRI
PET: positron emission tomography
CT: computerized tomography
GEP: Gene expression profiles
RT-PCR: real-time polymerase chain reaction
EGFR: Epidermal Growth Factor Receptor family
HER-2: human epidermal growth factor receptor 2
PDGF: platelet-derived growth factor
pRb: retinoblastoma
BRCA2: Breast Cancer susceptibility gene
PI3K-Akt/PTEN: Phosphatidylinositol 3-kinase- Protein kinase B/ Phosphatase and tensin homolog
Kiss-1: Kisspeptin1
PCR-SSCP: PCR- single-strand conformation polymorphism
VEGF: Vascular endothelial growth factor
CD: Cluster of differentiation
CSC: cancer stem cell
MMPs: matrix metalloproteinases
OCT4: octamer-binding transcription factor-4
HGFR or MET: Tyrosine-protein kinase or hepatocyte growth factor receptor
ALK: Anaplastic lymphoma kinase
ROS1: proto-oncogene receptor tyrosine kinase
RT: radio therapy
